# Supplementary material for: Sorting at embryonic boundaries requires high heterotypic interfacial tension
Source: Nat Commun. 2017 Jul 31;8:157. doi: 10.1038/s41467-017-00146-x (PMC5537356; doi:10.1038/s41467-017-00146-x)
Supplement: Supplementary file 2 — Supplementary Software 1 [file 41467_2017_146_MOESM2_ESM.zip › PottsModel/SrcPottsModel/doc/model/PerimeterEnergyFunction.html]

PerimeterEnergyFunction


JavaScript is disabled on your browser.


Skip navigation links


- Overview
- Package
- Class
- Use
- Tree
- Deprecated
- Index
- Help

- Prev Class
- Next Class

- Frames
- No Frames

- All Classes

- Summary:
- Nested |
- Field |
- Constr |
- Method

- Detail:
- Field |
- Constr |
- Method


model

## Class PerimeterEnergyFunction

- java.lang.Object
- - model.PerimeterEnergyFunction

- All Implemented Interfaces:
  :   EnergyFunction

  ---

    

  ```
  public class PerimeterEnergyFunction
  extends java.lang.Object
  implements EnergyFunction
  ```

- - ### Nested Class Summary

    - ### Nested classes/interfaces inherited from interface model.EnergyFunction

      `EnergyFunction.Energy`
  - ### Constructor Summary

    Constructors

    Constructor and Description

    `PerimeterEnergyFunction()`
  - ### Method Summary

    All Methods Static Methods Instance Methods Concrete Methods

    | Modifier and Type | Method and Description |
    | --- | --- |
    | `static double[]` | `getCurrentCellPerimeters(Pixel pSource, Pixel pTarget)` |
    | `double` | `getDeltaEnergy(Pixel s, Pixel t)` |
    | `EnergyFunction.Energy` | `getEnergyType()` |
    | `static double[]` | `getFutureCellPerimeters(Pixel pSource, Pixel pTarget)` |
    | `java.lang.String` | `getName()` |

    - ### Methods inherited from class java.lang.Object

      `equals, getClass, hashCode, notify, notifyAll, toString, wait, wait, wait`

- - ### Constructor Detail


    - #### PerimeterEnergyFunction

      ```
      public PerimeterEnergyFunction()
      ```
  - ### Method Detail


    - #### getEnergyType

      ```
      public EnergyFunction.Energy getEnergyType()
      ```

      Specified by:
      :   `getEnergyType` in interface `EnergyFunction`


    - #### getName

      ```
      public java.lang.String getName()
      ```

      Specified by:
      :   `getName` in interface `EnergyFunction`


    - #### getDeltaEnergy

      ```
      public double getDeltaEnergy(Pixel s,
                                   Pixel t)
      ```

      Specified by:
      :   `getDeltaEnergy` in interface `EnergyFunction`


    - #### getCurrentCellPerimeters

      ```
      public static double[] getCurrentCellPerimeters(Pixel pSource,
                                                      Pixel pTarget)
      ```


    - #### getFutureCellPerimeters

      ```
      public static double[] getFutureCellPerimeters(Pixel pSource,
                                                     Pixel pTarget)
      ```


Skip navigation links


- Overview
- Package
- Class
- Use
- Tree
- Deprecated
- Index
- Help

- Prev Class
- Next Class

- Frames
- No Frames

- All Classes

- Summary:
- Nested |
- Field |
- Constr |
- Method

- Detail:
- Field |
- Constr |
- Method
